# Transcription factor ZBTB42 is a novel prognostic factor associated with immune cell infiltration in glioma

**DOI:** 10.3389/fphar.2023.1102277

**Published:** 2023-01-25

**Authors:** Yanwen Li, Yongwei Zhu, Long Chen, Shunjin Xia, Abraham Ayodeji Adegboro, Siyi Wanggou, Xuejun Li

**Affiliations:** ^1^ Department of Neurosurgery, Xiangya Hospital, Central South University, Changsha, China; ^2^ Hunan International Scientific and Technological Cooperation Base of Brain Tumor Research, Xiangya Hospital, Central South University, Changsha, China

**Keywords:** glioma, ZBTB42, microenvironment, immune suppression, stemness

## Abstract

**Background:** ZBTB42 is a transcription factor that belongs to the ZBTB transcript factor family and plays an important role in skeletal muscle development. Dysregulation of ZBTB42 expression can lead to a variety of diseases. However, the function of ZBTB42 in glioma development has not been studied by now.

**Methods:** We analyzed the expression of ZBTB42 in LGG and GBM *via* the The Cancer Genome Atlas CGA and Chinese Glioma Genome Atlas database. Gene Ontology, KEGG, and GSVA analyses were performed to illustrate ZBTB42-related pathways. ESTIMATE and CIBERSORT were applied to calculate the immune score and immune cell proportion in glioma. One-class logistic regression OCLR algorithm was used to study the stemness of glioma. Multivariate Cox analysis was employed to detect the prognostic value of five ZBTB42-related genes.

**Results:** Our results show that ZBTB42 is highly expressed in glioma and may be a promising prognostic factor for Low Grade Glioma and GBM. In addition, ZBTB42 is related to immune cell infiltration and may play a role in the immune suppression microenvironment. What’s more, ZBTB42 is correlated with stem cell markers and positively associated with glioma stemness. Finally, a five genes nomogram based on ZBTB42 was constructed and has an effective prognosis prediction ability.

**Conclusion:** We identify that ZBTB42 is a prognostic biomarker for Low Grade Glioma and GBM and its function is related to the suppressive tumor microenvironment and stemness of glioma.

## Introduction

Glioma is the most lethal cancer among brain tumors which have a complex pathogenetic mechanism and characteristic that is prone to relapse (Taga and Tabu, 2020). WHO (World Health Organization) classified glioma as grades I to IV based on histopathological characteristics and prognostic factors. Glioblastoma (GBM) is the most aggressive, invasive, and malignant brain tumor and has been defined as grade IV by WHO (Hanif et al., 2017). There is no effective strategy to cure this malignant disease. After surgery and radiotherapy with concomitant temozolomide treatment, 5-year survival in patients with glioblastoma is only 4.1% (Stupp et al., 2009). One reason is the intricate tumor microenvironment (TME) in glioma. In addition to tumor cells, the TME also harbors stromal cells, extracellular proteins, chemokines, growth factors, etc. These stromal cells and extracellular matrix can facilitate tumor proliferation and help tumor cells resist hypoxia, radiotherapy, and chemotherapy (Nijkamp et al., 2013; Wu and Dai, 2017). In the meanwhile, the chemokines, cytokines, and growth factors secreted by tumor and stromal cells can induce immune cell infiltration in solid glioma tissue. The immune cells are usually reprogrammed into immunosuppressive phenotype and regulate the interaction between host and tumor, which can promote glioma development (Gieryng et al., 2017; Quail and Joyce, 2017). On the other hand, the immune checkpoints such as the programmed cell death 1 (PD-1) and the Cytotoxic T-lymphocyte associated protein 4 (CTLA4) expressed on the surface of the immune cells can decrease the T cell activation and proliferation (Huang et al., 2020). Therefore, immune therapy and immune checkpoint inhibitor (ICI) therapy have drawn much attention and brought hope to glioma patients.

ZBTB (Zinc finger and BTB domain-containing) transcript factors are a family of members, which is highly conserved in mammals and plays a crucial role in the development of the hemopoietic system and central neural system (Okado, 2021). Many ZBTB family genes such as Bcl6(ZBTB27), PLZF (ZBTB16), and Rp58 (ZBTB18, ZNF238), regulate neuronal cell’s fate lineage decision, migration, maturation, and maintenance (Tiberi et al., 2012; Xiang et al., 2012; Gaber et al., 2013). Whereas, deregulation of these genes promotes multiple kinds of tumor progression, especially glioma. Bcl6 and cofactor NCoR complex repress the MEK-ERK and S6K-RPS6 pathway *via* regulating the expression of AXL to promote glioma proliferation (Xu et al., 2017). PLZF can stimulate cellular transformation and proliferation in glioma and increase tumor growth by repressing the transcription of CDKN1A (Choi et al., 2014). Low expression of Rp58 is associated with the epithelial-mesenchymal transition (EMT) and cell survival in glioma (Tatard et al., 2010; Xiang et al., 2021).

Here, we find that ZBTB42, a member of the ZBTB transcription factor family, is a new biomarker for glioma. ZBTB42 is known to be expressed in skeletal muscle and testis and mutation of ZBTB42 leads to Lethal congenital contracture syndrome (LCCS) (Takahashi et al., 2008; Patel et al., 2014). ZBTB42 expression knockdown with shRNA in glioma cells induced decreased growth ability (Xu et al., 2017). More interestingly, ZBTB42 is almost never expressed in the normal brain while highly expressed in glioma tissue, but its mechanism of regulating glioma progression is still unknown. In this study, we found abnormally high expression of ZBTB42 in glioma and verified this discovery with clinical glioma samples and cultured cells. Then we demonstrated that increased expression of ZBTB42 leads to an immunosuppressive microenvironment and a worse prognosis, and ZBTB42 is highly related to immune checkpoint genes. On the other hand, glioma patients with high expression of ZBTB42 usually comprise higher stemness of glioma which may be another aspect of ZBTB42 potential function in glioma.

## Materials and methods

### Data collection

The transcriptome expression of glioma, LGG and GBM was downloaded from the TCGA data portal (https://tcga-data.nci.nih.gov/tcga/) and CGGA database (http://www.cgga.org.cn/). The patients without clinical information were excluded. The expression of ZBTB42 in pan-cancer and GTEx was downloaded from GEPIA2. The mRNA expression and methylation of ZBTB42 from the TCGA database and GSE databases were obtained from the Brainbase website tool.

### Clinical tissue collection and cell culture

15 glioma tissues and 11 normal brain samples were collected from Xiangya Hospital, Central South University between January 2016 and January 2022. Gliomas were classified according to 2016 WHO classification: five WHO II cases, four WHO III cases, and six WHO IV cases. The glioma tissues of different WHO grades and normal brain tissues were analyzed by immunohistochemistry staining (IHC) and 11 pairs of glioma samples and normal brain samples were analyzed by RT-qPCR. This study was approved by the Ethics Committee of Xiangya Hospital of Center South University. Human glioma cells HA 1,800, A172, U87, U251, HS683, and LN229 were purchased from Shanghai Cell bank. All cells were cultured in a humidified atmosphere containing 5% CO_2_/95% air at 37°C. Dulbecco’s Modified Eagle’s Medium (high glucose) with 10% fetal bovine serum (Bovogen) and 1% penicillin/streptomycin was applied to culture cells.

### Real-time quantitative polymerase chain reaction

The samples were kept at −80°C freezer until RNA extraction. We used Total RNA Extractor (Sangon Biotech, China) to extract RNA from clinical samples and cultured cells. The Prime Script® RT reagent Kit (Takara) was applied to synthesize RNA into cDNAs. RT-qPCR was performed in the 7,500 Real-time PCR System (Applied Biosystems) with SYBR Premix Ex Taq (Takara, Japan). The primers are ZBTB42: 5′-GCCGCCTACTGGACTTCATGTAC-3′(Forward), 5′-GCC​CTT​GCA​GAC​CTT​GAC​GAT​G -3′(Reverse) and GAPDH: 5′- TGACATCAAGAAGGTGGTGAAGCAG-3′(Forward), 5′-GTGTCGCTGTTGAAGTCAGAGGAG-3′(Reverse). Each assay was carried out in triplicate and 2^-△△Ct^ was calculated to analyze the gene expression difference.

### Immunohistochemistry

Glioma tissues of different WHO grades and normal brain tissues were fixed with 4% paraformaldehyde and embedded in paraffin. Then, the tissues were sectioned into 4 µm and rehydrated with gradient concentration ethanol. Citrate buffer was used for antigen retrieval and 3% hydrogen peroxide (H_2_O_2_) was applied to quench endogenous peroxidase. After blocking in 10% normal goat serum, the sections were incubated with ZBTB42 antibody (1:500, HPA, HPA066961) overnight at 4°. Then, the sections were incubated by secondary antibody (goat anti-rabbit IgG, 1:5,000, Proteintech) for 1 h at room temperature. Finally, the sections were stained with diaminobenzidine tetrahydrochloride (DAB) and hematoxylin. The quantification of ZBTB42 immunohistochemistry staining was performed by the software ImageJ.

### Gene set enrichment analysis and protein-protein interaction (PPI) network

The Differential Expression Genes (DEGs) were generated by “limma” package from the high ZBTB42 expression group and low expression group. The Gene Ontology (GO) and Kyoto Encyclopedia of Genes and Genomics (KEGG) enrichment were then performed *via* the R package “clusterProfiler.” The GSVA Reactome and Hallmark gene sets were obtained from the Molecular Signatures Database (MSigDB). The PPI was generated from the website tools STRING (https://string-db.org/).

### Immune-related analysis

ESTIMATE algorithms were applied to calculate the immune score, stromal score, and ESTIMATE score of the high ZBTB42 expression and low expression groups. The proportion of immune cell infiltration was generated by “CIBERSORT.” Correlation analysis of ZBTB42 with immune-related genes and stemness signature genes was carried out by R package “corrplot.”

### Statistical analysis

All statistical analyses were performed on R studio version 4.2.0. The Wilcoxon rank-sum test was applied to analyze the expression of ZBTB42 in cultured cells, AOD of normal tissue and glioma samples, and different clinicopathological subgroups. All statistical tests were two-sided. The *p* < .05 was regarded as a significant difference. The optimal cutting point was determined by the R package “Survminer” to separate glioma, LGG, and GBM patients into high ZBTB42 expression and low expression groups. The Kaplan-Meier plotter was utilized to illustrate the overall survival of glioma, LGG, and GBM patients between the high ZBTB42 expression and low expression groups. Least Absolute Shrinkage and Selection Operator (LASSO) regression filtrated DEGs between the high ZBTB42 expression and low expression groups into five prognostic genes. Multivariate cox regression analysis was performed to detect the independent prognostic performance of these genes. The nomogram based on prognostic genes was constructed by R package “rms.” The area under curve (AUC) was generated by the R package “survivalROC” to evaluate the predictive ability of the model.

## Result

### ZBTB42 expression analysis in pan-cancer and glioma

**FIGURE 1:**
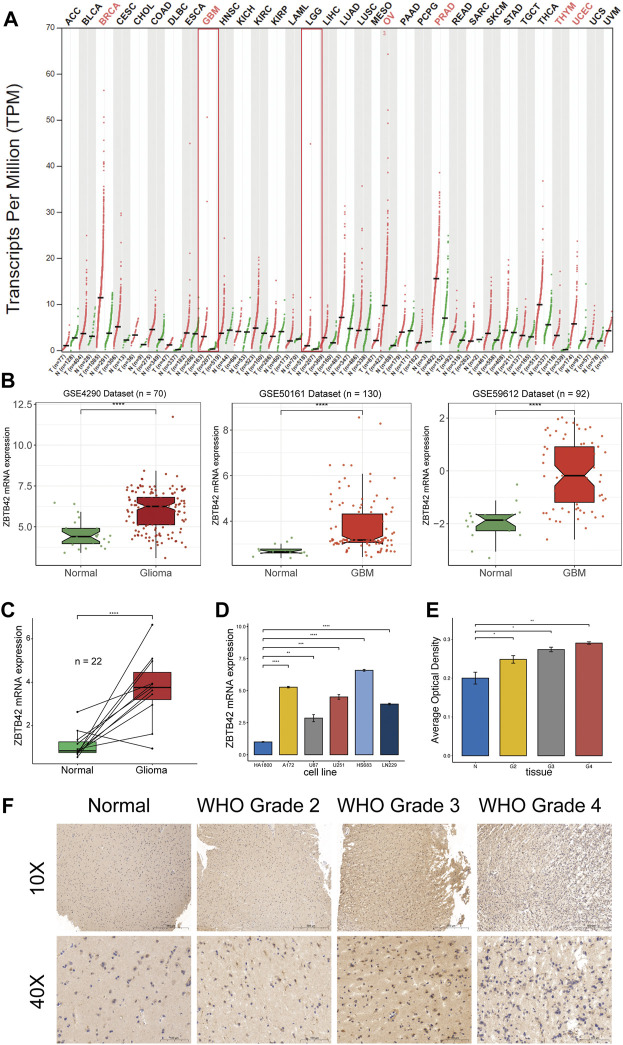
ZBTB42 expression profile in pan-cancer and glioma. **(A)** ZBTB42 mRNA expression of pan-cancer in TCGA dataset. **(B)** ZBTB42 is highly expressed in glioma compared with normal tissue in three different GEO datasets. **(C)** ZBTB42 mRNA expression in normal brain and glioma tissues. **(D)** ZBTB42 mRNA expression in human glia cell line and different glioma cell lines. **(E)** Quantification of ZBTB42 IHC staining between different WHO grades of gliomas and normal tissues (*n* = 12). **(F)** Immunohistochemistry staining of ZBTB42 in normal brain and glioma samples (*n* = 12). *p < 0.05; **p < 0.01; ***p < 0.001; ****p < 0.0001.

To investigate the expression of ZBTB42 in normal tissue and tumors, we used the online tool GEPIA2 to analyze this gene in 34 tumors versus adjacent tissues (or GTEX). According to Figure 1A, the expression of ZBTB42 is slightly increased in breast invasive carcinoma (BRCA), glioblastoma multiforme (GBM), ovarian serous cystadenocarcinoma (OV), Prostate adenocarcinoma (PRAD), thymoma (THYM), and uterine corpus endometrial carcinoma (UCEC), while it is also increased in many other tumors including Brain Low Grade Glioma (LGG). To further verify ZBTB42 expression in glioma, especially in GBM, Brainbase was used to analyze multiple glioma GSE datasets. In GSE4290, GSE50161, and GSE59612, ZBTB42 expression is highly elevated in glioma and GBM (Figure 1B). Furthermore, we performed ZBTB42 RT-qPCR on glioma and normal brain tissue. The increased expression of ZBTB42 was observed and the difference was significant (Figure 1C). Compared with normal human glia cells, the A172, U87, U251, HS683, and LN229 shows increasing expression of ZBTB42 (Figure 1D). The immunohistochemistry staining showed an evident ZBTB42 signal in different WHO grades of glioma samples (Figure 1F). The quantification analysis of area optical density (AOD) indicated that ZBTB42 is higher expressed in glioma tissues compared with normal brain tissue (Figure 1E). The graphic schematic and immunofluorescence on the U-2 OS and MCF7 cell lines showed that ZBTB42 is expressed in the nucleus and cell membrane (Supplementary Figures S1A, B). Meanwhile, we found that in the GTEx dataset, ZBTB42 is lowly expressed in brain tissue (Supplementary Figures S1C, D) which indicated that ZBTB42 may play a role in the development of glioma.

### ZBTB42 shows expression preference in malignant subtypes of glioma and is correlated with tumor progression

**FIGURE 2:**
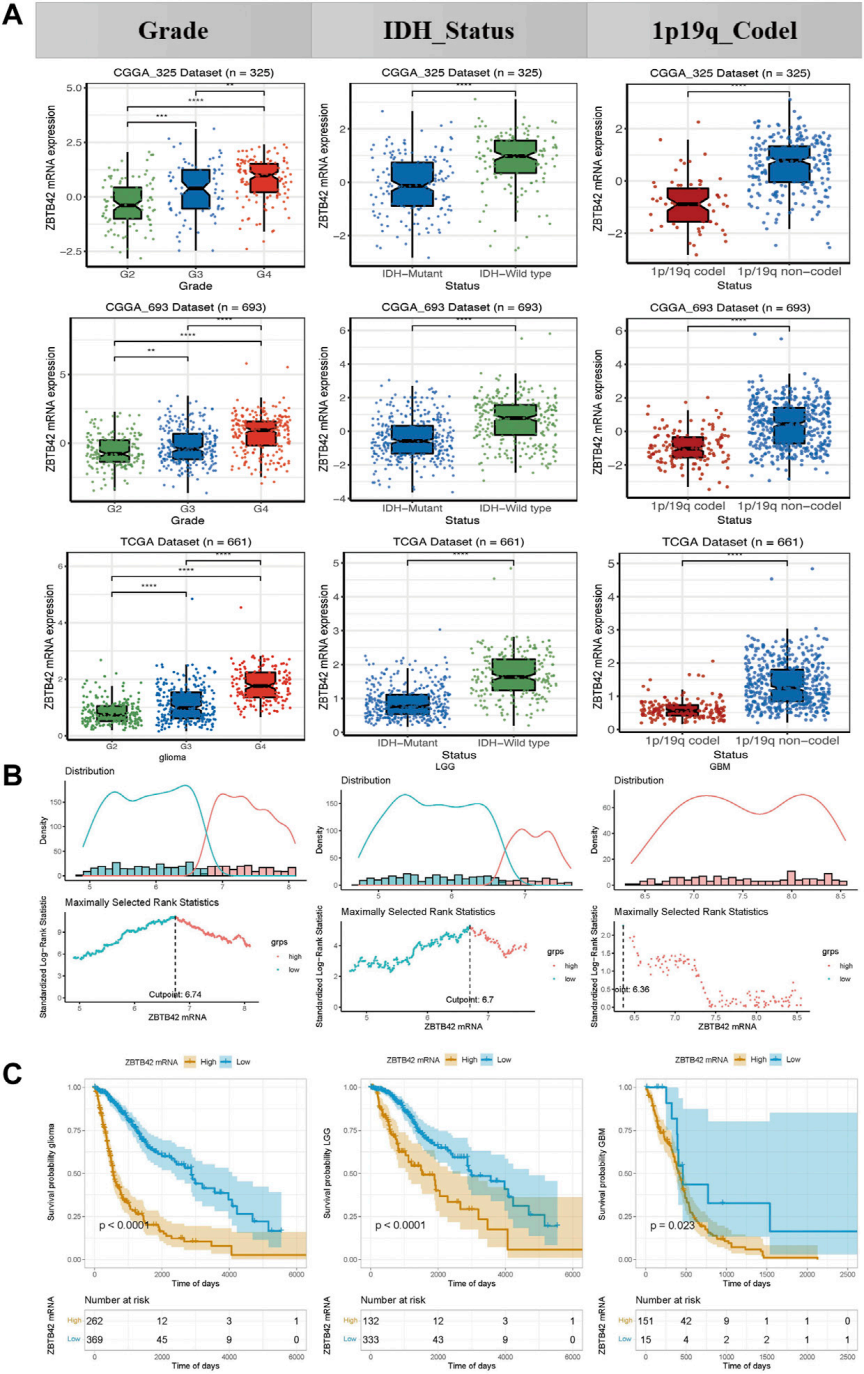
ZBTB42 expression in different subgroups of glioma and correlated with tumor progression. **(A)** Expression of ZBTB42 in clinical subgroups of glioma. **(B)** Optimal cut point determination in glioma, LGG, and GBM. **(C)** High expression of ZBTB42 leads to poor prognosis in both LGG and GBM. *p < 0.05; **p < 0.01; ***p < 0.001; ****p < 0.0001.

To further understand the distribution of ZBTB42 in glioma with different clinical parameters, we analyzed the glioma patients from the CGGA-325 cohort, CGGA-693 cohort, and the TCGA dataset by the Brainbase website. Interestingly, the level of ZBTB42 expression increased with the improvement of the WHO grade in all glioma datasets (Figure 2A). Remarkably, compared with the IDH mutant and 1p/19q codeletion subgroup, a higher expression of ZBTB42 was observed in the IDH wild type and 1p/19q non-codeletion subgroup (Figure 2A). These data suggested that ZBTB42 may be involved in glioma malignancy progression. Then we asked does ZBTB42 deregulation plays a role in the progression of glioma. We divided the 631 TCGA glioma patients into high ZBTB42 expression and low expression groups by optimal cutoff point (Figure 2B). Kaplan-Meier plotter analysis showed that the patients in the high expression group, have poor overall survival (Figure 2C). When we analyzed the LGG and GBM separately, the conclusions were the same (Figure 2C).

**FIGURE 3:**
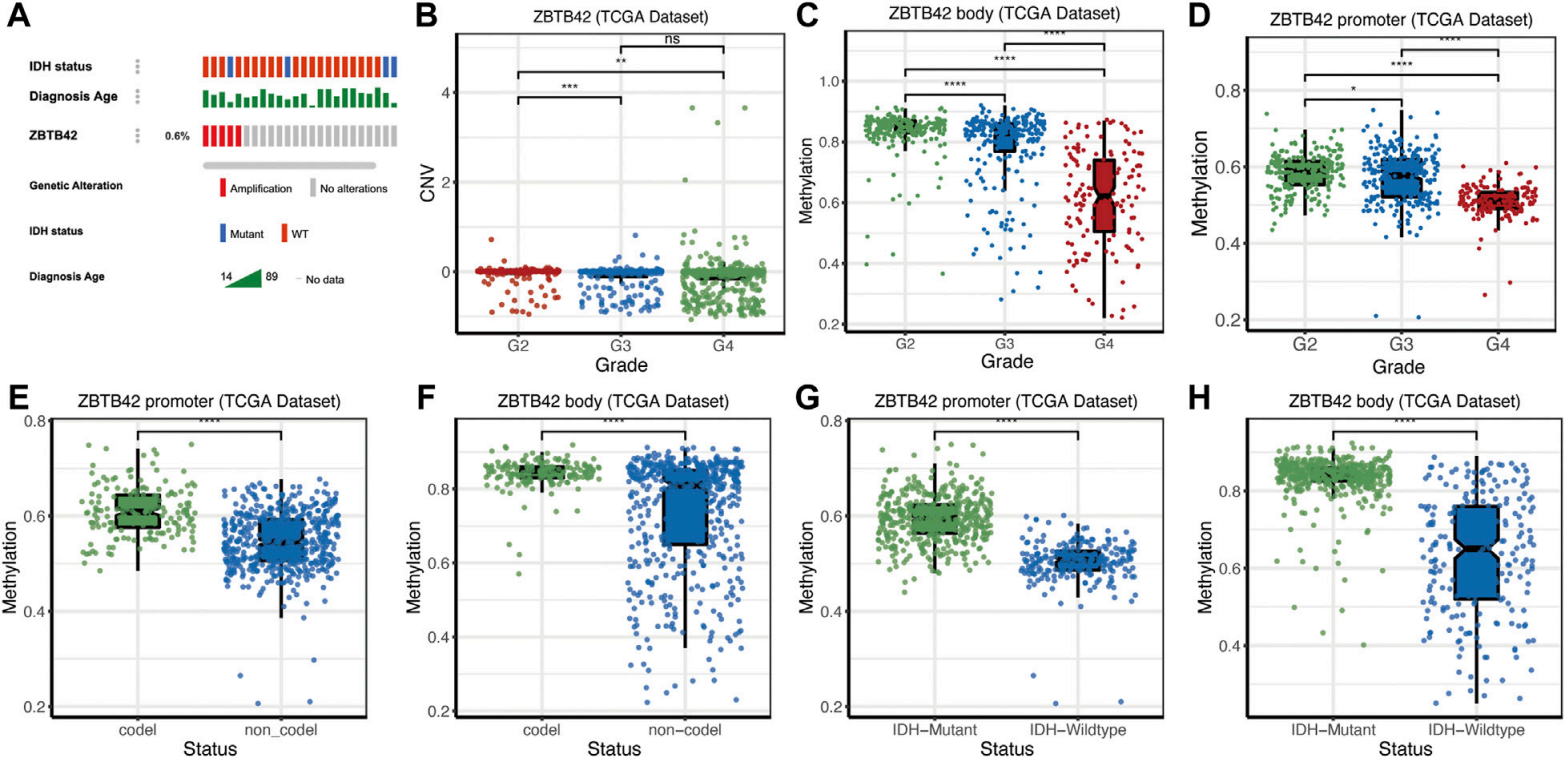
The copy number variations, mutation and epigenetic modification of ZBTB42 **(A)** Amplification state of ZBTB42 in glioma. **(B)** Copy number variations (CNVs) changes of ZBTB42. **(C,D)** Methylation of ZBTB42 promoter and ZBTB42 body in different WHO grades glioma. **(E,F)** Methylation of ZBTB42 promoter and ZBTB42 body in 1p19q codel and 1p19q non-codel subgroups. **(G,H)** Methylation of ZBTB42 promoter and ZBTB42 body in IDH wild type and mutant subgroups. *p < 0.05; **p < 0.01; ***p < 0.001; ****p < 0.0001.

To answer why the expression of ZBTB42 is elevated, we moved to the genetic alterations, copy number variation (CNV), and methylation modification of ZBTB42 in the TCGA dataset. Firstly, we check the genetic alterations state. ZBTB42 is amplified in .6% of glioma patients and most of which are IDH wild-type glioma (Figure 3A). The CNV decreased in Grade 3 glioma compared with Grade 2 while there was no significant difference between Grade 3 and Grade 4 (Figure 3B). In terms of epigenetics, we found that the methylation of ZBTB42 promoter and body decreases accompanied by Grade 2 to Grade 4 (Figures 3C, D). A similar demethylation of ZBTB42 also appeared in the IDH wild-type and 1p19q non-codeletion subgroup (Figures 3E–H). Therefore, the demethylation of the promoter and body may result in the increased expression of ZBTB42, and abnormal expression of ZBTB42 reveals a more severe tumor progression.

### Pathway enrichment analysis of dysregulation of ZBTB42 in TCGA cohort

**FIGURE 4:**
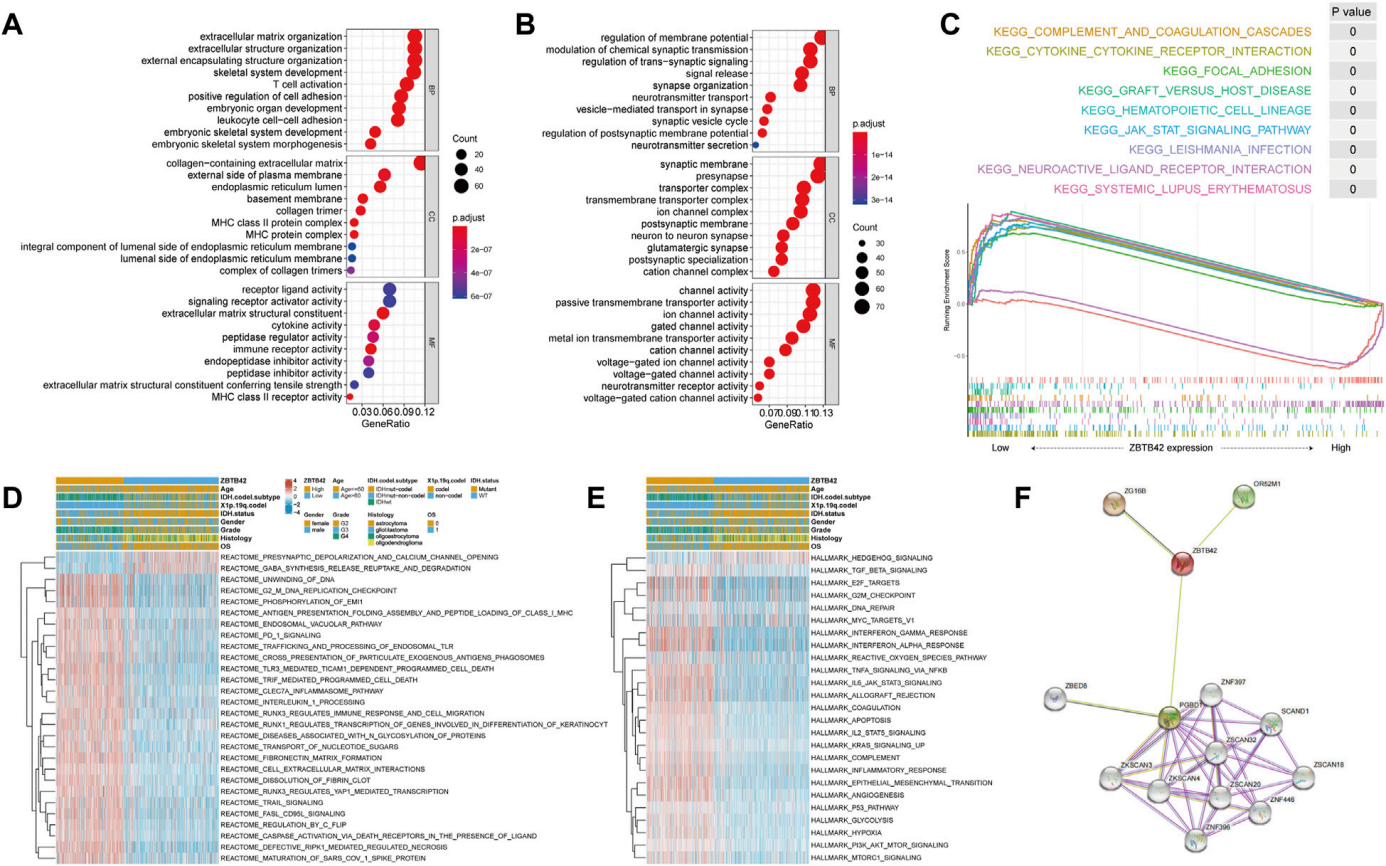
Pathway enrichment analysis of dysregulation of ZBTB42 in TCGA cohort. **(A,B)** Biological process analysis on upregulated genes **(A)** and downregulated genes **(B)** between the ZBTB42 high expression group and low expression group. **(C)** The pathways related to upregulated genes and downregulated genes by KEGG enrichment analysis. **(D)** GSVA analysis on Reactome gene set between glioma patients with high ZBTB42 expression and low expression. **(E)** GSVA analysis on Hallmark gene set between glioma patients with high ZBTB42 expression and low expression. **(F)** Protein-protein interaction network based on ZBTB42-related genes by STRING database. The lines between genes represent protein-protein associations and different colors represent how these relationships were validated. The green line represents two genes are neighborhood genes; The pink line represents the relationship that has been experimentally determined by existing papers. The black line represents two genes that are co-expressed. The purple line represents protein homology.

To elucidate the effect of ZBTB42 alteration on biological functions in glioma, we compared the high ZBTB42 expression group and low expression group in glioma and filtered out upregulated and downregulated genes. After that, GO and KEGG enrichment analyses were performed. In GO analysis of upregulated genes, we found that except for skeletal system development which we already know, most related pathways were focusing on extracellular matrix and immune-related pathways such as T cell activation, MHC class II protein complex, and immune receptor activity (Figure 4A). Spearman correlation analysis showed that ZBTB42 was highly related to the T cell activation-related genes, such as CD28, CD247, AKT1, etc., (Supplementary Figure S2). In terms of downregulated genes, the pathways were focusing on the channel activity of the cell member (Figure 4B). In the KEGG analysis, the upregulated genes were in the hematopoietic cell lineage, cytokine-cytokine receptor, and JAK-STAT signaling pathway (Figure 4C). Taken all together, we speculated that dysregulation of ZBTB42 in glioma affects glioma progression *via* the tumor microenvironment, especially the immune microenvironment.

To verify this hypothesis, we performed GSVA analysis with DEGs between the high ZBTB42 expression group and low expression group on Reactome and Hallmark gene sets from MSigDB. The GSVA Reactome analysis suggests multiple pathways such as PD1 signaling, CLEC7A inflammasome, and immune response were positively related to upregulated genes. Besides that, the cell-extracellular matrix, cell cycle, and cell death pathways were also highly related to these genes (Figure 4D). In GSVA hallmark analysis, a similar result was detected (Figure 4E), which confirmed the relativity between ZBTB42 and the glioma microenvironment. In addition, protein-protein interaction suggested that ZBTB42 potentially interacted with PGBD1, ZSCAN20, and ZNF396. These genes are all associated with glioma prognosis and ZSCAN20 is related to the immune infiltration of tumors (Figure 4F).

### The high ZBTB42 expression group is associated with immune suppression in glioma

**FIGURE 5:**
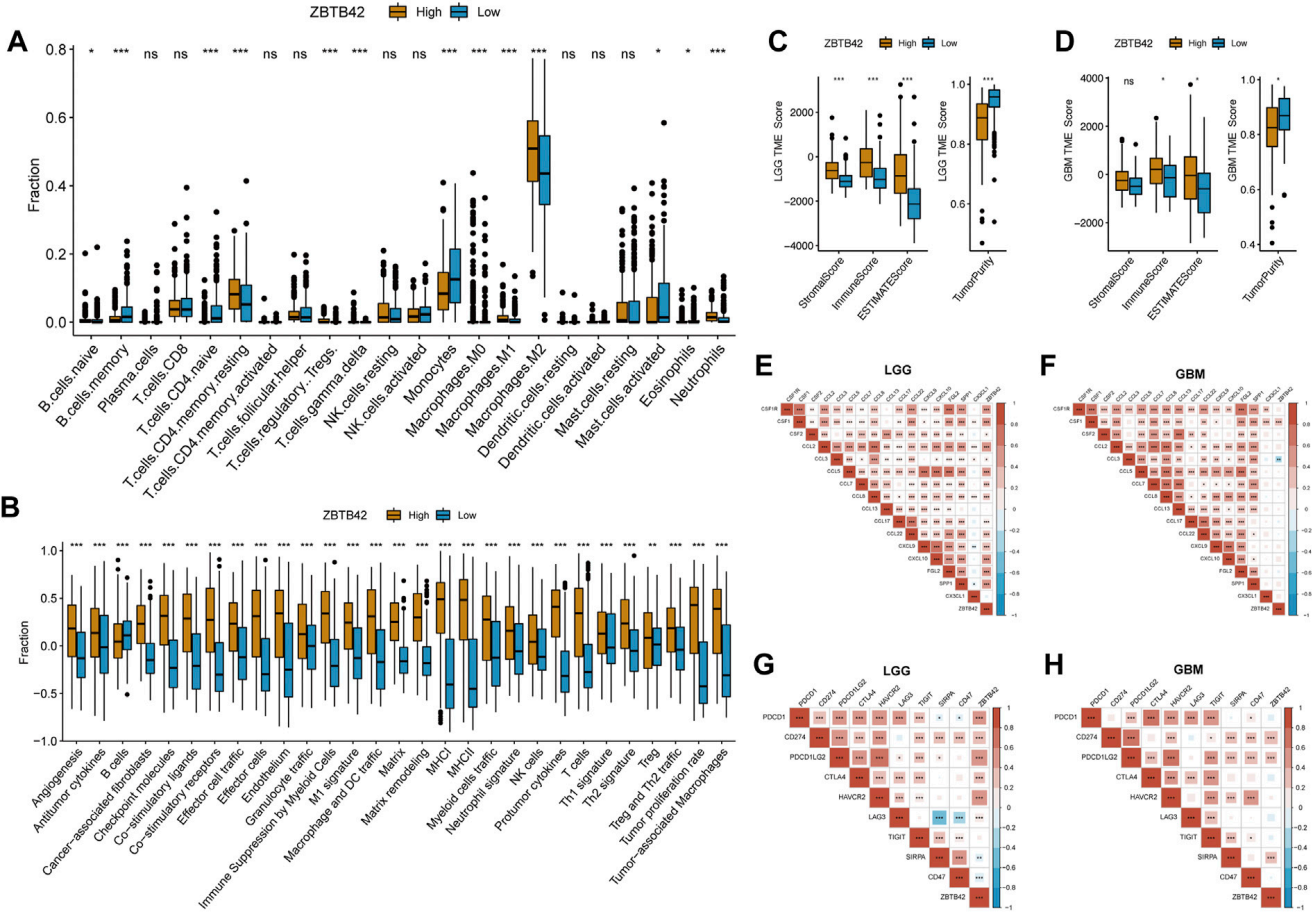
High ZBTB42 expression group is associated with immune suppression in glioma. **(A)** The fraction of 22 immune cell infiltration in high ZBTB42 expression group and low expression group of gliomas. **(B)** Immune-related signature scores between high ZBTB42 expression group and low expression group of gliomas by ssGSEA analysis. **(C,D)** Stromal score, Immune score, and ESTIMATE score, and tumor purity of LGG **(C)** and GBM **(D)** in the TCGA dataset. **(E–H)** Map of ZBTB42 correlation with cytokine genes, chemokine genes, and immune checkpoint genes in LGG **(E, G)** and GBM **(F, H)**. *p < 0.05; **p < 0.01; ***p < 0.001.

To further investigate the interaction between the high ZBTB42 expression group with the immune microenvironment in glioma, the CIBERSORT algorithm was applied to detect immune cell proportion in glioma from the TCGA dataset. Interestingly, compared with the low expression group, the high expression group had more immune cell infiltration, such as resting CD4^+^ memory T cells, Treg cells, M1 Macrophages, and M2 Macrophages (Figure 5A). In contrast, the number of memory B cells, naïve T cells, and monocytes in the high expression group decreased. Increasing T reg cells and M2 macrophages suggested that there was immune suppression in the high expression group microenvironment. Then, we performed ssGSEA analysis to explore the immune-related signature variations. The results showed that checkpoint molecules, immune suppression by myeloid cells, protumor cytokines, and Treg signature were increased in the high expression group (Figure 5B). To calculate immune scores and verify the presence of infiltrating immune cells, ESTIMATE algorithms were employed in LGG and GBM patients from the TCGA dataset. In LGG, the result showed that all three scores were increased and the tumor purity was decreased in the high expression group, predicting the existence of more stroma cells and immune cells (Figure 5C). In GBM, the Stromal score, Immune score, and ESTIMATE score were higher and the tumor purity was lower in the high ZBTB42 expression group. However, the difference in stromal score wasn’t significant (Figure 5D). In addition, we found that ZBTB42 was positively related to chemokines and cytokines, such as FGL2, SPP1, and CCL, CXCL subfamilies (Figure 5E). In both LGG and GBM, ZBTB42 was positively related to CSF1, which is known for promoting glioma immune suppression (Figures 5E, F). On the other hand, the immune checkpoint gene like PD1 was reported to inhibit T cell effects, induce T cell inactivity and make T cells exhausted. Correlation analysis showed that ZBTB42 was positively related to PD1, PD-L1, PD-L2, CTLA4, TIM3, and LAG3 in LGG (Figure 5G) and positively related to PD-L1, PD-L2, SIRPA in GBM (Figure 5H), suggesting that the high expression of ZBTB42 may promote glioma progression *via* immune suppression microenvironment.

### High ZBTB42 expression is related to the stronger tumor-stemness feature of glioma

**FIGURE 6:**
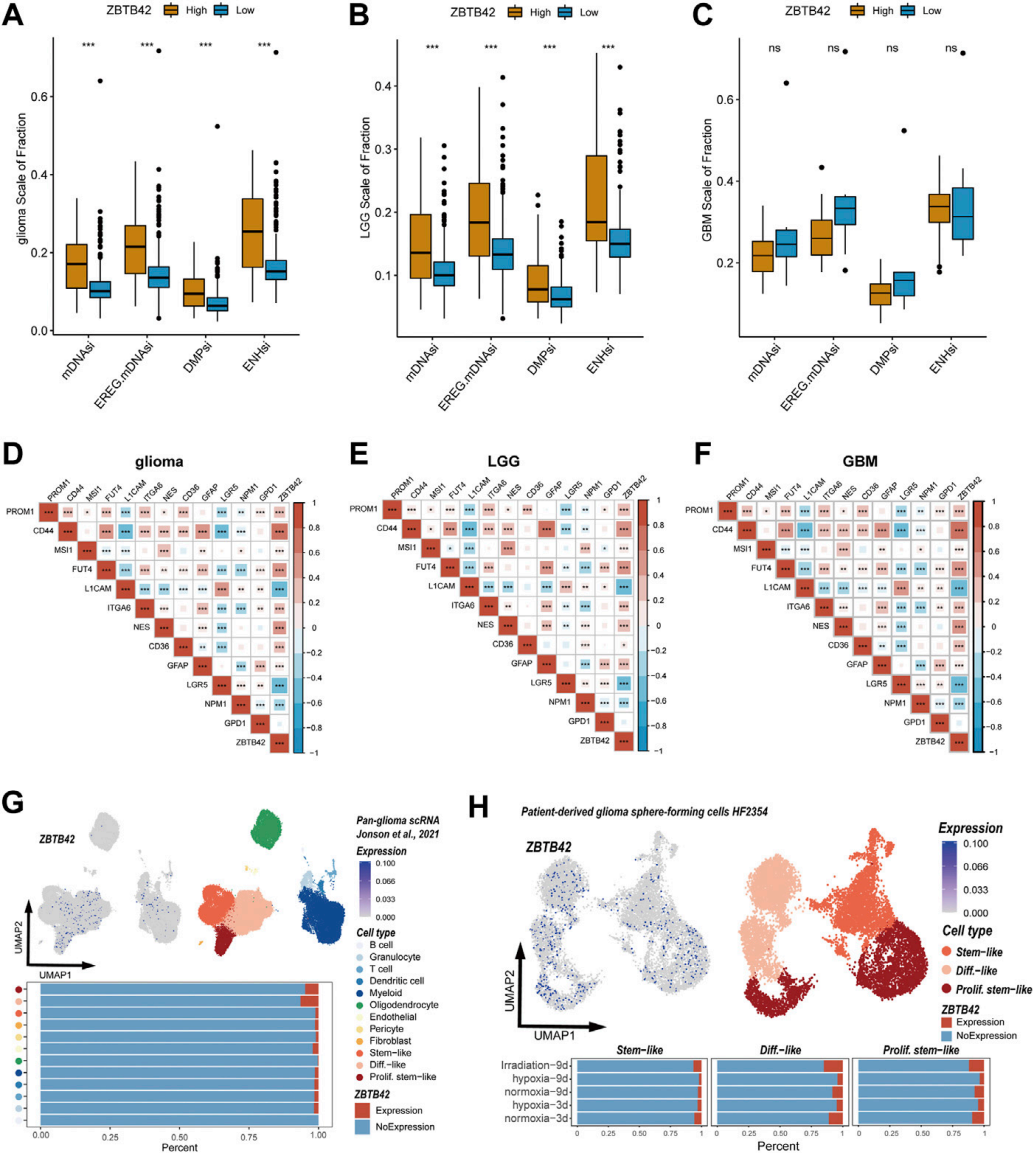
High ZBTB42 expression is related to the stronger tumor-stemness feature of glioma. **(A,B)** mDNAsi, EREG.mDNAsi, DMPsi, and ENHsi of high ZBTB42 expression and low expression group in glioma **(A)**, LGG **(B)** and GBM **(C)**. **(D–F)** Map of ZBTB42 correlation with stemness characteristic genes in glioma **(D)**, LGG **(E)**, and GBM **(F)**. **(G,H)** ZBTB42 was expressed in stem-like and proliferation stem-like cell subtypes analyzed by single cell sequencing data. *p < 0.05; **p < 0.01; ***p < 0.001.

Interestingly, in the GO and KEGG analysis, the upregulation genes were enriched in the cell cycle, and cytokines (Figure 4A). In ssGSEA analysis, tumor proliferation-related signatures and extracellular matrix signatures such as angiogenesis, protumor cytokines, tumor proliferation rate, and matrix remodeling were also significantly improved in the high ZBTB42 expression group (Figure 5B). Therefore, we asked if ZBTB42 is related to the stemness of glioma. To answer this question, four stemness indices were calculated by the one-class logistic regression (OCLR) algorithm in glioma, LGG, and GBM. We found that the stemness indices of the high expression group were significantly higher than the low expression group in glioma and LGG (Figures 6A, B). However, we didn’t get the same conclusion in GBM (Figure 6C). To further validate our assumption, correlation analysis was performed between ZBTB42 and stemness markers. ZBTB42 was positively related to stem cell markers like PROM1, CD44, MSI1, FUT4, ITGA6, NES, CD36, and GFAP in glioma, LGG, and GBM (Figures 6D–F). In addition, we collected the single cell sequence data of glioma. As a result, we found ZBTB42 was mainly expressed in stem-like cells and differentiation-like cells (Figure 6G). We also detected the expression of ZBTB42 in sphere-forming cells which received radiation and a hypoxia culture environment. Interestingly, the expression of ZBTB42 was increased in stem-like, differentiation-like, and proliferation stem-like cells after radiation, indicating that ZBTB42 wasn’t only related to the stemness of glioma but also may play a role in radiation resistance in glioma treatment (Figure 6H). These data suggested that increased expression of ZBTB42 was also associated with the stemness of glioma and may play a role in glioma stem cells.

### Construction of a ZBTB42-related prognostic model

**FIGURE 7:**
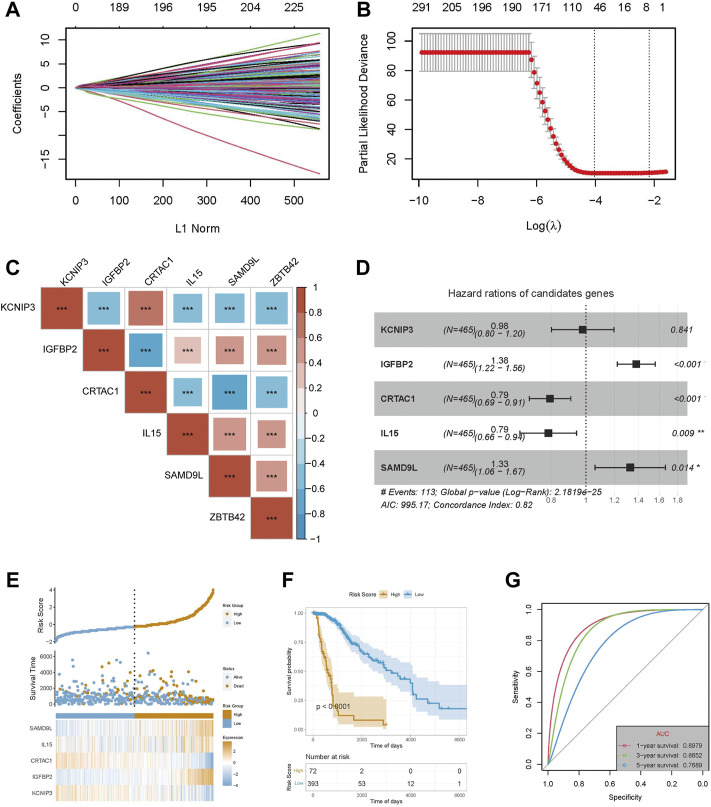
Construction of a prognostic model with ZBTB42-related genes in LGG. **(A)** LASSO coefficients profiles of DEG between high ZBTB42 expression group and low expression group in LGG. **(B)** LASSO regression with cross-validation obtained optimal prognostic-related genes in LGG. **(C)** Map of ZBTB42 correlation with prognostic related genes in LGG. **(D)** Multivariate Cox analysis of KCNIP3, IGFBP2, CRTAC1, IL15, and SAMD9L with clinical outcomes for LGG. **(E)** The risk score, survival time, and expression distribution of the five genes in the LGG cohort. **(F)** Kaplan-Meier survival analysis of high-risk model and low-risk model. **(G)** Prediction sensitivity validation of the prognostic model by receiver operating characteristic (ROC) curve analysis in 1, 3, and 5 years for LGG patients. *p < 0.05; **p < 0.01; ***p < 0.001.

To further illustrate the potential role of ZBTB42 in glioma, we applied Lasso regression on the DEGs between the high ZBTB42 expression group and low expression group in LGG patients (Figures 7A, B). 5 genes were detected which were mostly related to the clinical prognosis. Interestingly, all these genes were related to the expression of ZBTB42 in LGG (Figure 7C). Multivariate Cox analysis confirmed that these genes were independent prognostic factors for LGG patients (Figure 7D). The risk score and survival time showed that the high risk group had a poor clinical outcome (Figure 7E). Kaplan-Meier plotter analysis showed that the low-risk group of the patients had a better prognosis (Figure 7F). The area under the curve (AUC) of 1 year, 3 years, and 5 years were 0.898, 0.865, and 0.769 indicating that this model can predict the survival of glioma patients efficiently (Figure 7G). Furthermore, we verified the prognostic value of these genes in GBM and glioma *via* multivariate Cox analysis (Supplementary Figures S3A, B). The result suggested that KCNIP, IGFBP2, IL5, and SAMD9L were independent poor prognostic factors for GBM and glioma. On the other hand, CRTAC1 was associated with good clinical outcomes. The patients were divided into high risk group and low risk group based on the distribution of expression of five genes and the high risk group was associated with a bad prognosis (Supplementary Figures S3C, D, G, H). The Kaplan-Meier plotter and ROC analysis confirmed the good performance of these genes in the clinical prediction (Supplementary Figures S3E, F, I, J). The same analysis was also performed in the CGGA-325 cohort and CGGA-693 and the results support that 5 ZBTB42-related genes have good prognostic prediction ability (Supplementary Figure S4).

## Discussion

ZBTB42 was found in the testes, and regulates the development of skeletal muscle, while its function in tumors hasn’t been well described (Takahashi et al., 2008). Here we first illustrated the ZBTB42 expression profile in pan-cancer and investigated its potential relationship with glioma. High expression is detected in glioma and leads to a poor prognosis. The epigenetic modification of glioma plays a crucial role in tumor cell plasticity and resistance to hypoxia, chemotherapy, and radiotherapy (Johnson et al., 2021). The most common epigenetic alteration in malignant tumors is methylation. The methylation of intergenic regions, gene bodies, and DNA repetitive sequences in DNA repair and tumor suppressor genes is an important part of tumor formation and progression (Aoki and Natsume, 2019; Ehrlich, 2019). In glioma, the methylation status of the O6-methylguanine-DNA methyltransferase (MGMT) promotor is associated with the response to temozolomide treatment (Aoki and Natsume, 2019; Mathur et al., 2020). Our data show increased ZBTB42 promotor and gene body methylation preference in benign subtypes of glioma, which is negatively related to ZBTB42 expression. In addition, a high level of ZBTB42 methylation leads to better overall survival in LGG patients. Collectively, ZBTB42 is a prognostic biomarker of glioma and the hypomethylation of ZBTB42 is, at least partly, the reason for the promotion of ZBTB42 expression.

In our present study, we performed GO, KEGG, and GSVA analysis on the DEGs. The pathways enriched are mainly focused on immune response, T cell activation, cytokines, and JAK-STAT signaling. The tumor microenvironment is a complicated cellular milieu constructed during tumorigenesis which consists of tumor cells, immune cells, stromal cells, and extracellular matrix molecules (Hanahan and Coussens, 2012; Senga, 2021). The immune cells such as macrophages, T cells, B cells, natural killer cells, dendritic cells, and myeloid-derived suppressor cells (MDSCs) interact with stromal cells, tumor cells, cytokines and decide the immune characteristics and tumor progression (Nagarsheth et al., 2017). The tumor-associatediated macrophages (TAMs), which contain M1 and M2 subgroups, take up the largest proportion of the immune cells and usually play an immunosuppressive role in microenviroment regulation (Grabowski et al., 2021). M1 macrophages are antineoplastic because of their enhanced antitumor inflammatory reactions and intrinsic phagocytosis function while M2 macrophages behave as immune-suppressor with immunosuppressive factors secretion and decreased antigen-presentingenting ability (Ruffell et al., 2014; Zhou et al., 2017; Liu et al., 2021). Besides the antiinflammation, the M2 macrophages can also induce angiogenesis to promote tumor growth and metastasis (Martinez et al., 2008; Fleetwood et al., 2009). In the tumor microenvironment, T reg cells are a subset of CD4^+^ T cells and they can curtail the function of multiple immune cells by decreasing the production of interleukin (IL)-2 and interferon (IFN)-γ, increasing Th2 cytokine skewing, and directly inhibiting of endogenous generation and expansion (Humphries et al., 2010). Our immune cell infiltration analysis shows increasing M2 macrophages and T-reg cells in the high ZBTB42 expression group. In the contrast, the number of memory B cells, naïve T cells, and monocytes decreased. Colony-stimulating factor-1(CSF-1) plays an important role in the differentiation and survival of TAM (Pyonteck et al., 2013). Several experiments were performed to target glioma-associated macrophage populations by colony-stimulating factor-1 receptor (CSF-1R). In mice, inhibition of CSF-1R can either block the transformation of M2 macrophages or deplete TAMs to prevent glioma progression and invasion (Yan et al., 2017). The survival in the preclinical model was enhanced efficiently in the treatment group (Pyonteck et al., 2013; Sun et al., 2019; Akkari et al., 2020). In the correlation analysis, ZBTB42 is positively related to the expression of CSF-1 in both LGG and GBM indicating ZBTB42 is associated with immune suppression in glioma and this feature may be related to the increased expression of CSF-1.

Moreover, the immune checkpoint genes can induce immune suppression and anti-immune checkpoint inhibitors (ICI) have been wildly studied both in basic research and clinical trials (Qi et al., 2020). Programmed Cell Death Protein 1 (PD-1), which has become the most comprehensively immune checkpoint molecule, is a transmembrane protein on the T and B cells and plays a crucial role in inducing immunosuppression. PD-1 can modulate the activity of T-cells, activate apoptosis of antigen-specific T cells, and inhibit apoptosis of Treg cells (Han et al., 2020). Cytotoxic T-lymphocyte-associated protein 4 (CTLA-4) express on the activated T cells and Treg cells and belongs to the immunoglobulin superfamily. CTLA4 can inhibit T cell co-stimulatory by combining with the ligands CD80 and CD86 which are expressed on antigen-presenting cells (APCs) (Fong et al., 2012). We found that ZBTB42 is associated with PD1, and PD-L1. PD-L2, CTLA4 HAVCR2, LAG3. Considering that ZBTB42 is expressed in the nucleus and cell membrane, targeting ZBTB42 may help people precisely kill cells with immune checkpoints and promote the overall survival of patients.

On the other hand, glioma stem cells (GSCs) are an important part of the glioma microenvironment and regulate glioma initiation, progression, and recurrence (Folkins et al., 2007). In the tumor microenvironment, the GSCs can secret cytokines such as fibroblast growth factor 2 (FGF2), hypoxia-inducing factor (HIF), and vascular endothelial growth factor (VEGF) to promote tumor invasion, recruit immune cells, induce angiogenesis, and self-renew (Hambardzumyan and Bergers, 2015). ZBTB42 is positively related to glioma stem cells marker genes such as CD44, MSI1, Fut4, and NES and the high expression group has a stronger relationship with glioma stemness. Interestingly, we found that ZBTB42 was expressed in the stem-like, proliferation stem-like, and differentiation-like cells based on the download single cell sequencing data (Johnson et al., 2021). After radiation, the percentage of ZBTB42 in the above tumor cells was increased, indicating that ZBTB42 may play a role in the radiation resistance of glioma cells.

Finally, we sorted out 5 genes which highly related to ZBTB42 and they showed potent prognostic value. Based on these genes, we constructed a nomogram model which has a sensitive prognosis prediction ability in LGG, GBM, and glioma patients. This model may help clinicians make clinical prognosis predictions and decide on treatment strategies.

## Conclusion

In summary, we have identified ZBTB42 as a novel prognostic biomarker for glioma. ZBTB42 is related to immune suppression and glioma stemness in the microenvironment. Targeting ZBTB42 treatment may help glioma patients have better overall survival.

## Data Availability

The original contributions presented in the study are included in the article/Supplementary Material, further inquiries can be directed to the corresponding authors.
