# Supplementary material for: Transcription factor ZBTB42 is a novel prognostic factor associated with immune cell infiltration in glioma
Source: Front Pharmacol. 2023 Jan 25;14:1102277. doi: 10.3389/fphar.2023.1102277 (PMC9905726; doi:10.3389/fphar.2023.1102277)
Supplement: Supplementary file 1 [file Presentation1.pdf]

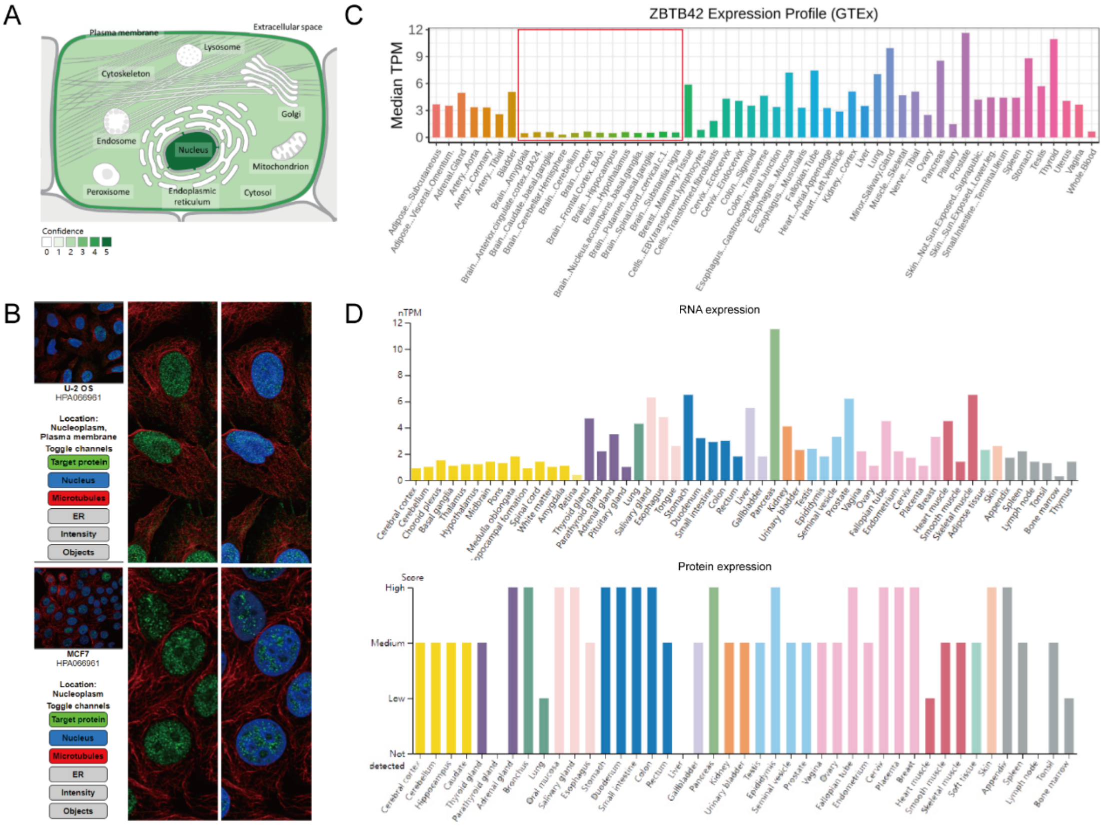

Fig.S1 The expression of ZBTB42 in normal tissue and different cell lines.

A. ZBTB42 expression profile in normal tissue from GTEx dataset.

B. RNA expression and protein expression of ZBTB42 in different organs from the Human Protein Atlas.

C. The graphic schematic of ZBTB42 expression generated from COMPARTMENTS subcellular localization database.

D. Immunofluorescence of ZBTB42 in U-2 OS and MCF7 cell lines.

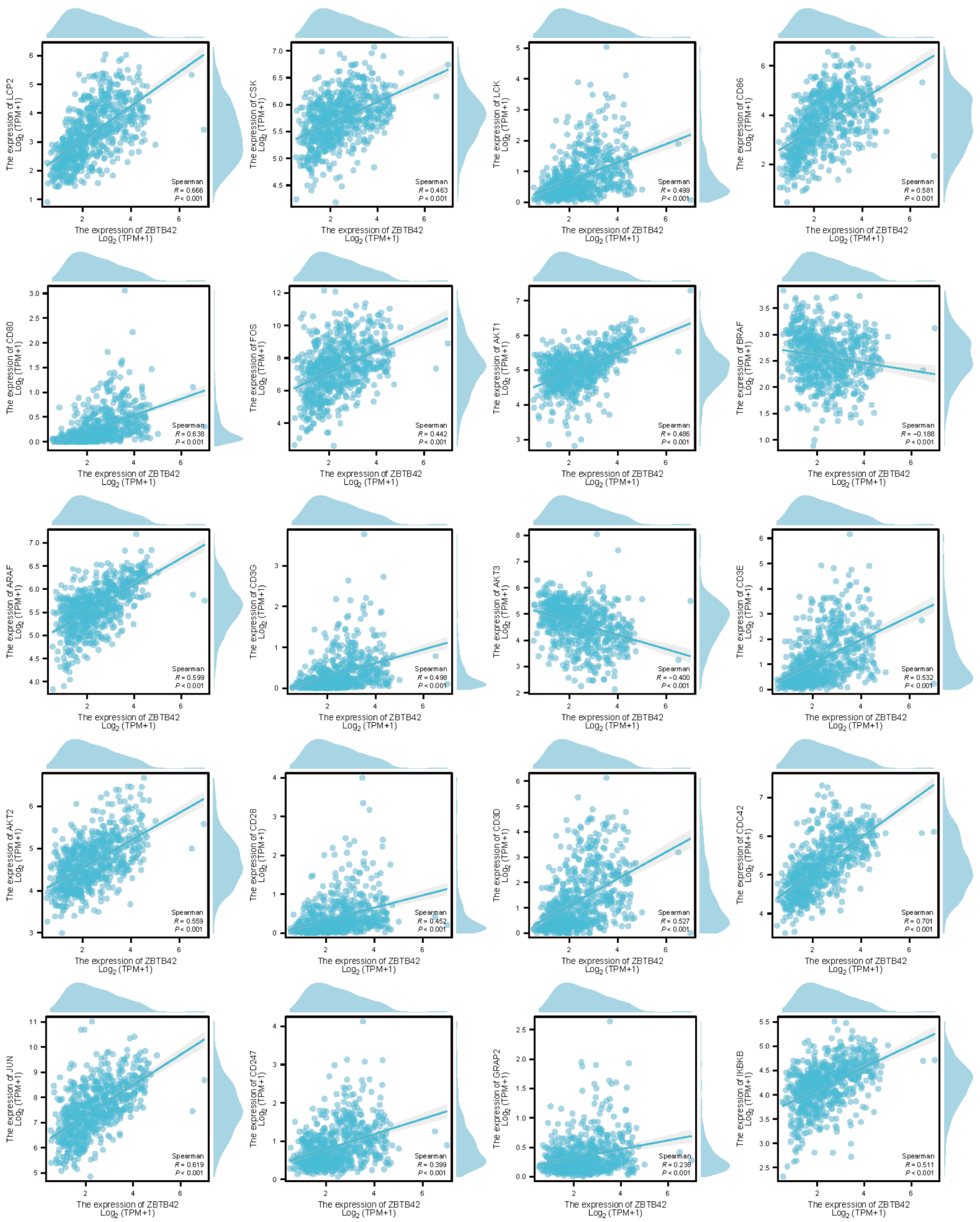

Fig.S2 Correlation analysis between ZBTB42 and T cell activation-related genes.

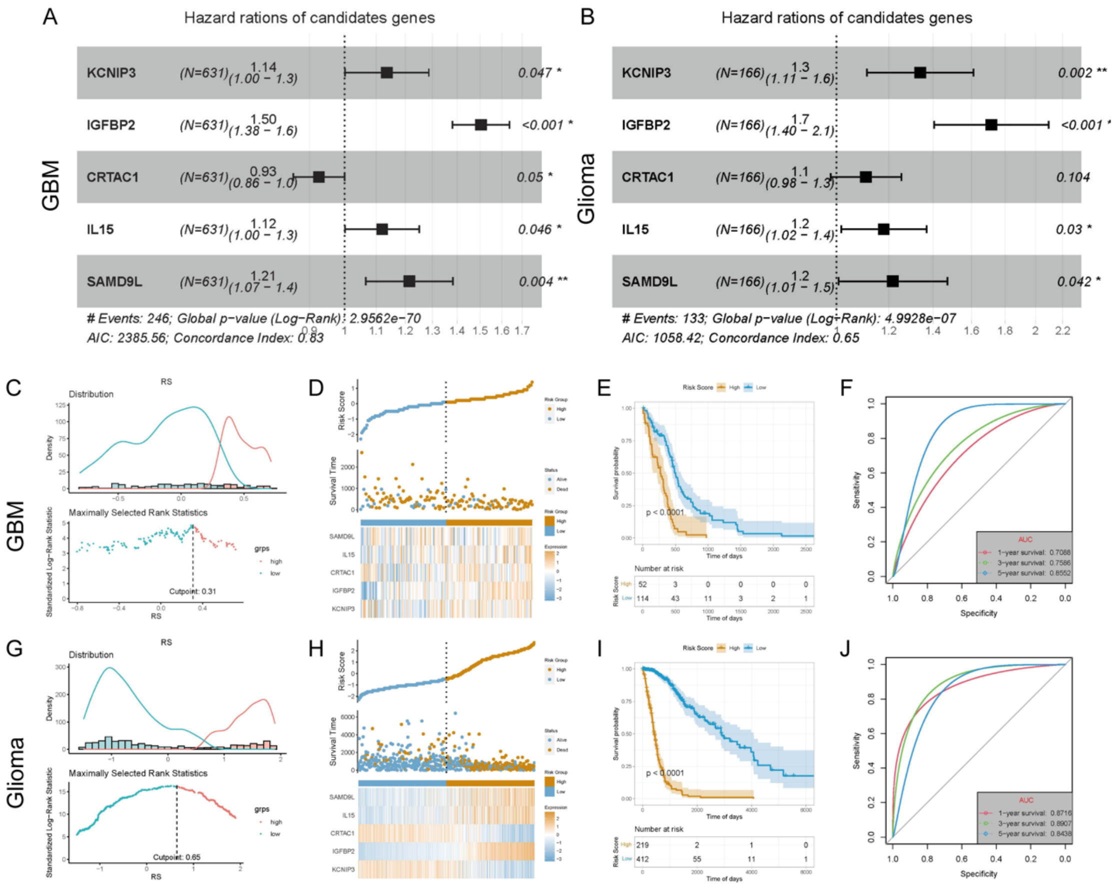

Fig.S3 Construction of a prognostic model with ZBTB42-related genes in GBM and glioma.

(A-B). Multivariate Cox analysis of KCNIP3, IGFBP2, CRTAC1, IL15, and SAMD9L with clinical outcomes for GBM and glioma. (C, G) The optimal cutoff points of ZBTB42 expression in GBM cohort(C) and glioma cohort(G). (D, H) The risk score, survival time, and expression distribution of the five genes in the GBM cohort(D) and glioma cohort(H). (E, I) Kaplan-Meier survival analysis of high-risk model and low-risk model in GBM cohort(E) and glioma cohort(I). (F, J) Prediction sensitivity validation of the prognostic model by receiver operating characteristic (ROC) curve analysis in 1, 3, and 5 years for GBM patients(F) and glioma patients(J).

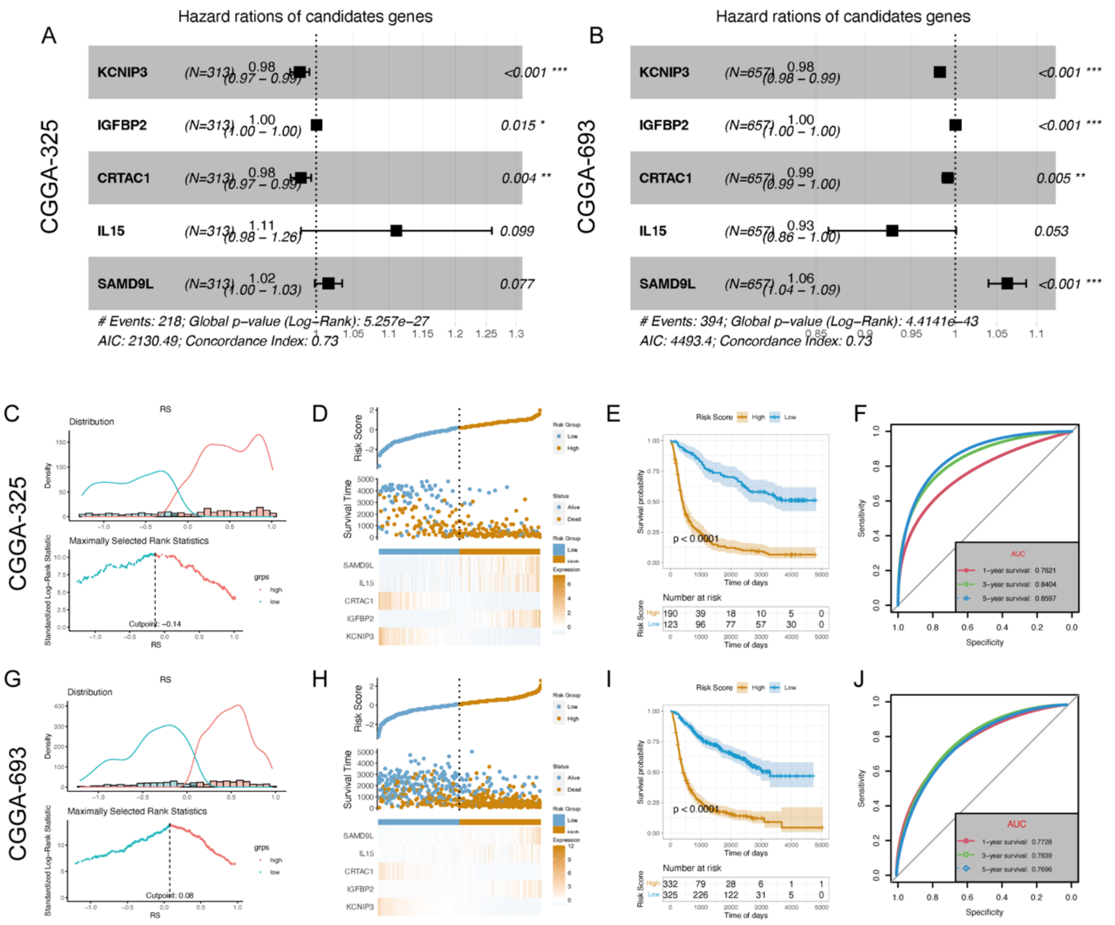

Fig.S4 Construction of a prognostic model with ZBTB42-related genes in CGGA glioma cohorts.

(A-B). Multivariate Cox analysis of KCNIP3, IGFBP2, CRTAC1, IL15, and SAMD9L with clinical outcomes for CGGA glioma cohorts. (C, G) The optimal cutoff points of ZBTB42 expression in CGGA-325 cohort(C) and CGGA-693 cohort(G). (D, H) The risk score, survival time, and expression distribution of the five genes in the CGGA-325 cohort(D) CGGA-693 cohort(H). (E, I) Kaplan-Meier survival analysis of high-risk model and low-risk model in CGGA-325 cohort(E) and CGGA-693 cohort(I). (F, J) Prediction sensitivity validation of the prognostic model by receiver operating characteristic (ROC) curve analysis in 1, 3, and 5 years for CGGA-325 cohort(F) and CGGA-693 cohort(J).
