# Supplementary figures and images for: Transcription factor ZBTB42 is a novel prognostic factor associated with immune cell infiltration in glioma
Source: Front Pharmacol. 2023 Jan 25;14:1102277. doi: 10.3389/fphar.2023.1102277 (PMC9905726; doi:10.3389/fphar.2023.1102277)

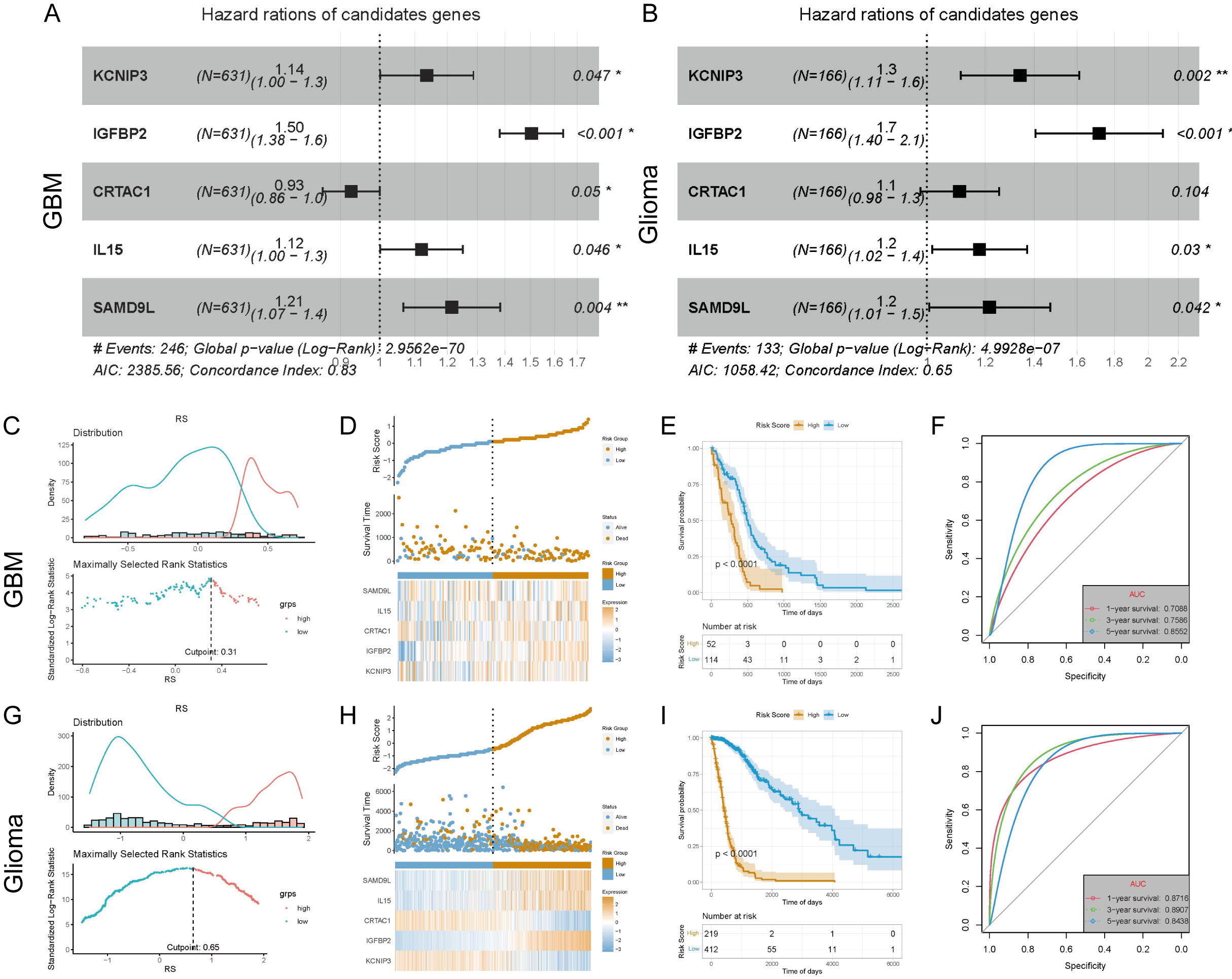

Supplement: Supplementary file 2 [file Image3.tif]

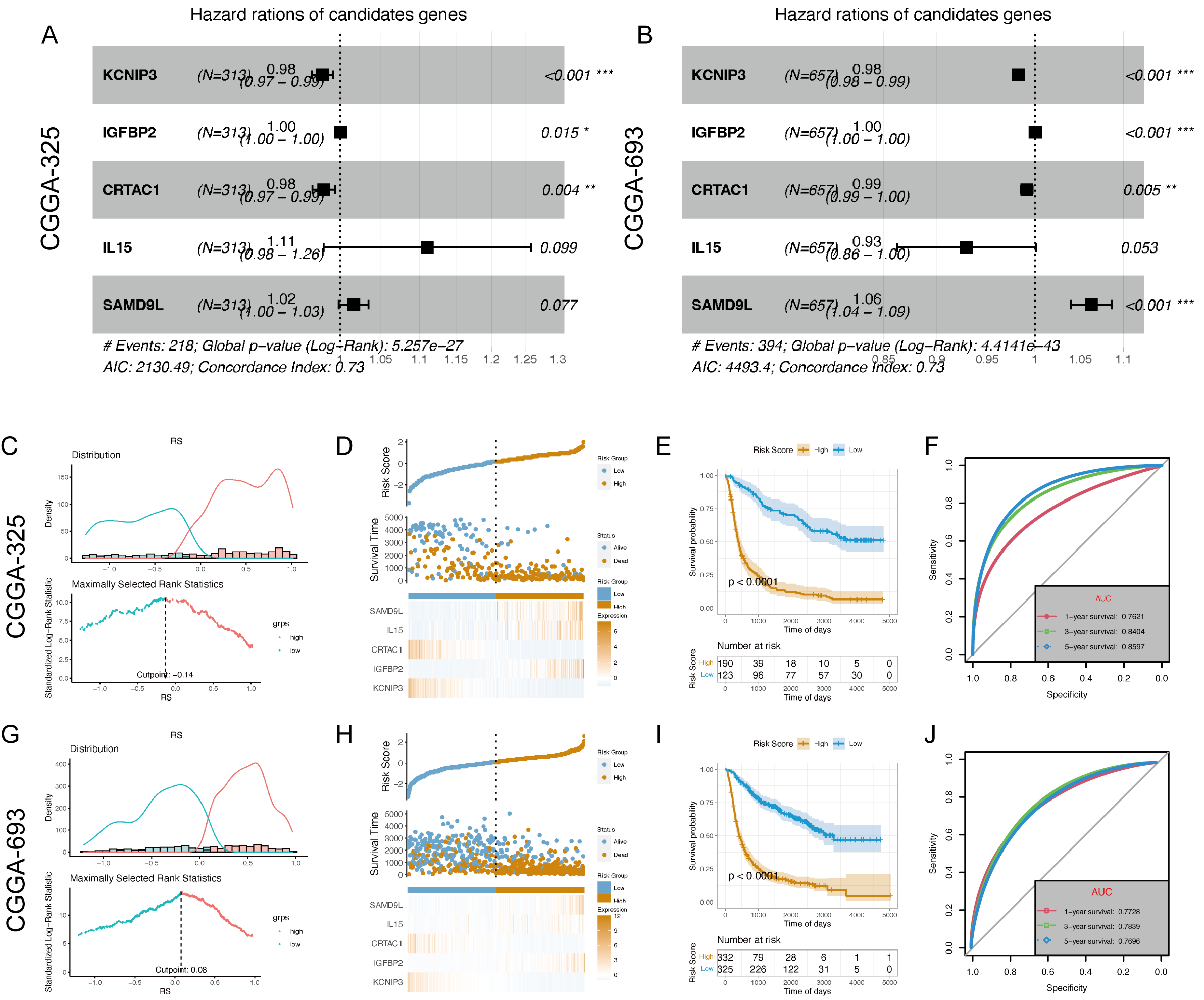

Supplement: Supplementary file 3 [file Image4.tif]

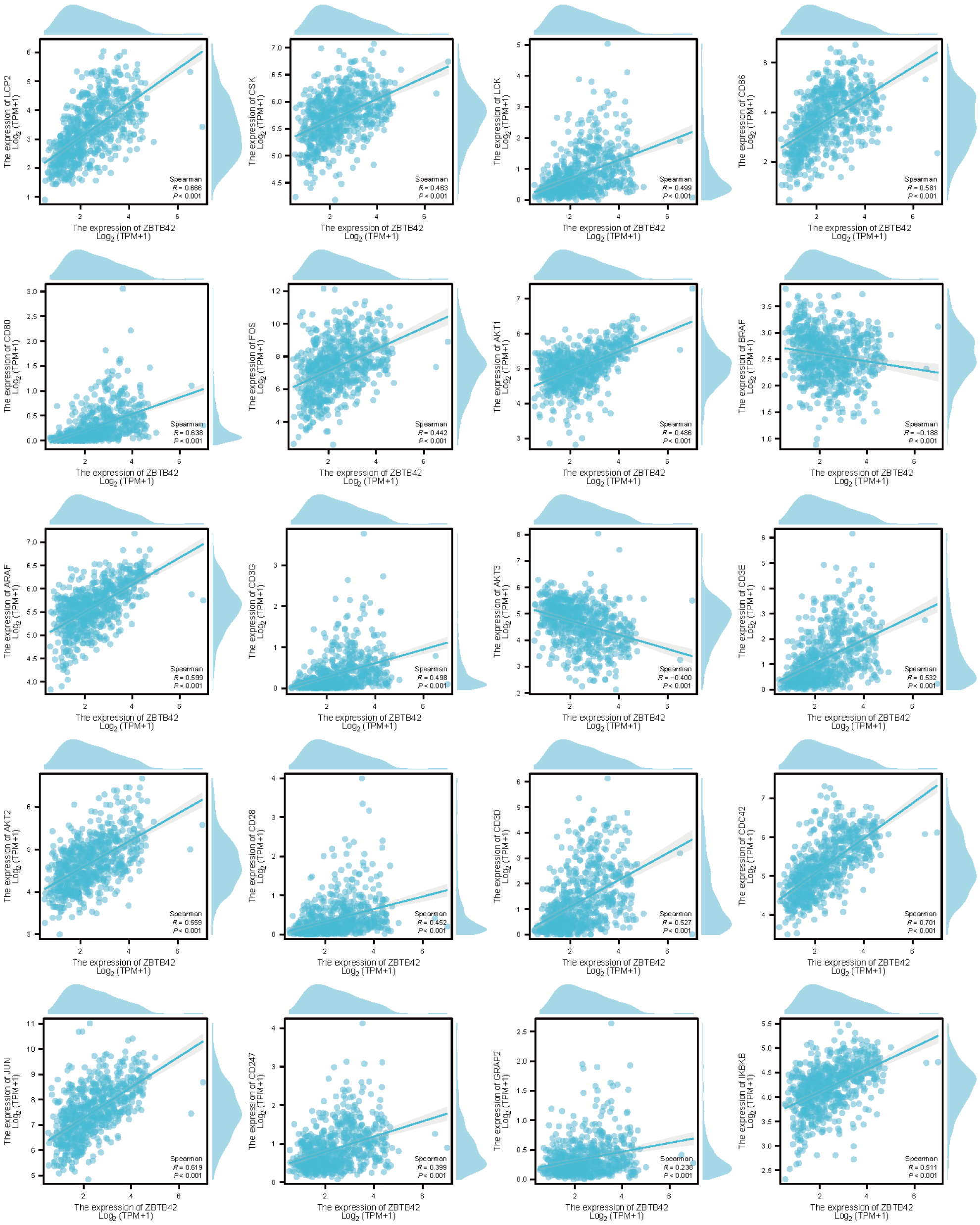

Supplement: Supplementary file 4 [file Image2.tif]

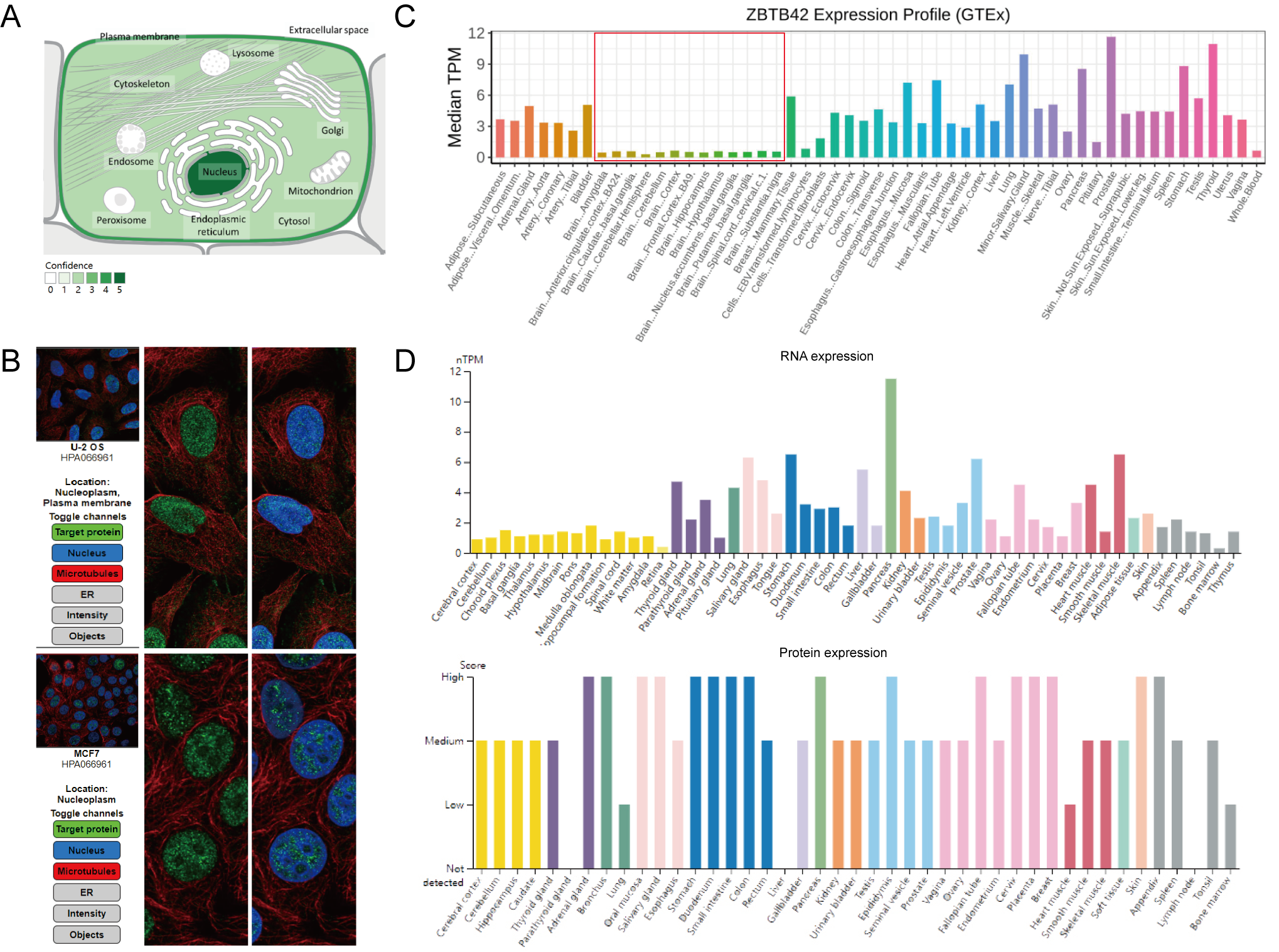

Supplement: Supplementary file 5 [file Image1.tif]
