# Supplementary material for: Transcription factor ZBTB42 is a novel prognostic factor associated with immune cell infiltration in glioma
Source: Front Pharmacol. 2023 Jan 25;14:1102277. doi: 10.3389/fphar.2023.1102277 (PMC9905726; doi:10.3389/fphar.2023.1102277)
Supplement: Supplementary file 6 [file Table2.DOCX]

**Data generated from Web tools.**

**1**. ZBTB42 pan-cancer expression data is generated by Web tools ---GEPIA

Link: http://gepia.cancer-pku.cn/detail.php?gene=ZBTB42

**2**. ZBTB42 expression data, methylation data in clinical subgroups, and mRNA expression of ZBTB42 in GTEx dataset are obtained from Web tools ---BrainBase.

Link: https://ngdc.cncb.ac.cn/brainbase/geneInfo/ZBTB42
